# Fully Bio-Based Adhesive from Tannin and Sucrose for Plywood Manufacturing with High Performances

**DOI:** 10.3390/ma15248725

**Published:** 2022-12-07

**Authors:** Guoming Xiao, Jiankun Liang, De Li, Yuan Tu, Bengang Zhang, Feiyan Gong, Wen Gu, Min Tang, Xinyue Ding, Zhigang Wu, Hong Lei

**Affiliations:** 1 College of Forestry, Guizhou University, Guiyang 550025, China; 2 College of Civil Engineering, Kaili University, Qiandongnan 556011, China; 3 Yunnan Provincial Key Laboratory of Wood Adhesives and Glued Products, Southwest Forestry University, Kunming 650224, China; 4 School of Chemistry and Material Engineering, Zhejiang A&F University, Hangzhou 311300, China

**Keywords:** green manufacturing for plywood, tannin, sucrose, fully bio-based wood adhesive

## Abstract

Fully bio-based adhesives are beneficial to reduce the dependence of the wood adhesive industry on synthetic resins based on petrochemical resources and enhance the market competitiveness of adhesives. A fully bio-based wood adhesive composed of tannin and sucrose was developed and successfully used in the preparation of plywood. Effects of the preparation technology on the bonding strength and water resistance of plywood were investigated, and the properties of the adhesive were analyzed by Fourier transform infrared spectroscopy (FT-IR), thermogravimetry (TG) and X-ray diffraction (XRD) in this study. The results showed that: (1) Compared with other biomass adhesives, tannin–sucrose adhesive had the characteristics of high-solid content and low viscosity, which had the potential to prepare particleboard and fiberboard. (2) A proper mass ratio of tannin to sucrose was key to obtaining a tannin–sucrose adhesive with better properties. (3) The optimum preparation process of tannin–sucrose adhesive for plywood was as follows: hot-pressing temperature of 210 °C, hot-pressing time of 1.2 min/mm, m(tannin):m(sucrose) of 60:40 and adhesive loading of 160 g/m^2^. Under these conditions, the water-resistant bonding strength of the plywood was 0.89 MPa, which met the strength requirements of the Type II standard of plywood in GB/T 17657-2013. (4) The hot-pressing temperature played a decisive role in the tannin–sucrose adhesive, and the good performance of the plywood was maintained when the temperature was 210 °C or above. Thus, the prepared tannin–sucrose adhesive had high-bonding strength, good water resistance and thermal stability.

## 1. Introduction

**Figure 1:**
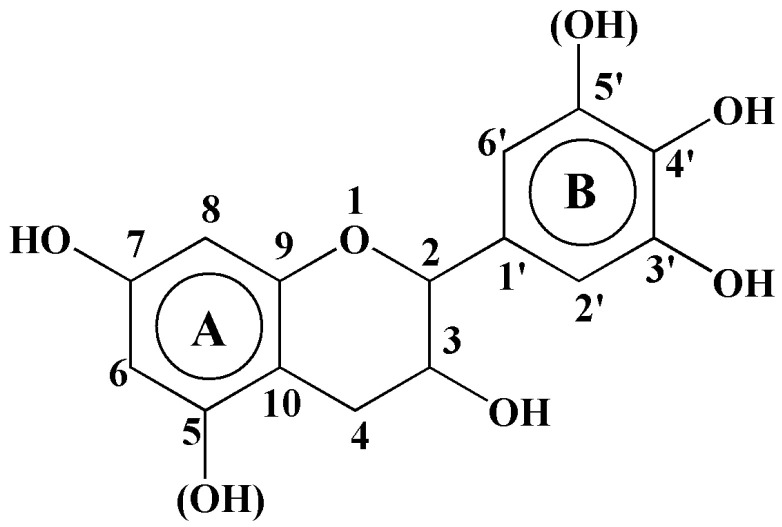
Unit structure of condensed tannins.

The adhesives used in the wood-based panel industry are predominantly formaldehyde-based adhesives, with the usage amount accounting for 60–70% or higher of the total adhesive usage [1,2,3,4]. Formaldehyde-based adhesives have a very prominent social and environmental disadvantage, namely, formaldehyde is released during adhesive preparation and use as well as the preparation of wood-based panels made with such adhesives, thus endangering the environment and human health [5,6,7]. With the continuously rising price of petrochemical products and the increasing enhancement in people’s environmental awareness, the development and application of bio-based adhesives have aroused increasing attention and related research reports into tannin-based adhesives [8,9], soy protein-based adhesives [10,11,12,13,14], lignin-based adhesives [15,16,17,18], etc. In early works, plywood produced using bio-adhesives had problems of low-bonding strength and poor water resistance. Subsequently, formaldehyde-based resins were used for modification. However, the introduction of formaldehyde, a toxic substance in formaldehyde-based resins, greatly reduced the environmental protection characteristics of biomass adhesives, at the cost of sacrificing its own environmental advantages, the pursuit of superior performance [19,20,21]. Therein, tannin-based adhesives have been most successfully investigated and applied and have slowly been applied in industrial production in some countries. According to chemical composition, tannin can be divided into hydrolysable tannin and condensed tannin, where the former is characterized by low yield and reactivity with formaldehyde and non-high polymer structures. Condensed tannin is the current focus of tannin utilization and one of the main materials studied for wood adhesives. Condensed tannin is mainly formed by polymerizing flavonoids in different bonding forms, such as C_4_-C_8_, C_4_-C_6_ and C_2_-*O*-C_7_. The B ring generally does not participate in reactions [22,23,24,25], so the reactivity of condensed tannins is mainly attributed by the reactivity of the A ring (Figure 1). According to the presence/absence of C_5_ site of the A ring, the structural units of condensed tannins can be divided into resorcinol-A ring type and phloroglucinol-A ring type.

With a polyphenolic structure similar to phenol, tannins can substitute partial or all phenols in phenol–formaldehyde resin adhesives and react with formaldehyde to act upon wood adhesives. Rightly based on this account, tannin-based adhesives constitute another application field of tannins in addition to the leather-making industry and have been successful applied to industrialized production in countries rich in tannin resources, such as South Africa.

Common tannin-based adhesives can be prepared very simply, only needing to add formaldehyde in the tannin solution before hot-pressing, but the prepared adhesives face various problems such as a low degree of crosslinking, low-bonding strength, and poor moisture resistance, which are mainly ascribed to their large molecular weight, specifically, the viscosity significantly increases and fluidity is lost if a there is a low polycondensation degree in the tannin–formaldehyde reactions, and consequently, distant reactive sites remaining in tannin molecules from each other, thus failing to form methylene bonds. To prepare environmentally friendly tannin-based adhesives, the environmental protection property of bond bridge growth catalysts is of great importance, mainly concentrating on epoxy resins, polyamides, isocyanates, etc. [26,27,28,29]. Although the mechanical properties and water resistance of modified tannin-based adhesives are considerably improved, these modifying agents are still based on petrochemical products, and if introduced in sufficient quantity, the original intention of the research on tannin-based adhesives is obviously violated, so they cannot be called pure-biomass environmentally friendly tannin-based adhesives.

Zhao reported a tannin–sucrose adhesive used to prepare particleboard [30,31,32]. One of the important processes for biomass transformation and the utilization of glucose lies in the sucrose-catalyzed conversion and synthesis of furan aldehydes, such as hydroxymethyl furfurals. Accordingly, it was thought that the successful preparation of tannin–sucrose adhesives could be derived from the reaction between tannin and hydroxymethyl furfurals. Tannins can be divided into hydrolysable tannins and condensed tannins, where the former is rich in polyphenolic acids, which are natural acids capable of forming H^+^ through ionization in aqueous solution. The main components of condensed tannins are flavanols and their derivatives, the former of which contain polyphenolic structures similar to phenols with more phenolic hydroxyl groups. Moreover, they can form H^+^ through ionization in aqueous solution so as to form relatively stable quinone structures, thus presenting acidic properties [33,34,35]. Hence, H^+^ ionized by tannins provides a certain acidic environment for the adhesive system, which contributes to the crosslinking reaction with sucrose. Although Zhao’s study fully indicated that tannin–sucrose adhesives could successfully prepare particleboard, plywood and particleboard were not totally the same in terms of the requirements for adhesives, including molecular weight, polycondensation degree, crosslinking density, adhesive loading, etc. On this basis, plywood was prepared using tannin–sucrose adhesives, and the feasibility of preparing the plywood with tannin–sucrose composite adhesive and the preparation technology were studied in this paper. In this study, the emphasis was to explore the effect of the tannin–sucrose mass ratio on the performances of tannin–sucrose adhesive, as well as an appropriate preparation technology for plywood, aiming to lay the foundations for the research and development of fully biomass-based wood adhesives.

## 2. Materials and Methods

### 2.1. Materials

Waxberry tannin (160 meshes, industrial grade) was obtained from Guangxi Wuming Tannin Extract Plant Co., Ltd. (Nanning, China) Sucrose (99.0%, analytically pure) was obtained from Chengdu Jinshan Chemical Reagents Co., Ltd. (Chengdu, China). Dodecylbenzene sulfonic acid (SDBS, >90.0%, analytically pure) was purchased from Tianjin Fengchuan Chemical Reagent Co., Ltd (Tianjin, China). Distilled water was made in the laboratory. Poplar veneer (moisture content of 8–10%), which was bought from Shuyang, Jiangsu, with a length × width of 400 mm × 400 mm and thickness of 1.5 mm.

### 2.2. Preparation of the Tannin–Sucrose Adhesives and Plywood and the Test of the Bonding Strength

At room temperature, distilled water, tannin and sucrose were added into a round-bottom three-mouth flask equipped with a mechanical stirring rod, a thermometer and a condenser pipe, and was stirred evenly. Subsequently, 0.3 g of SDBS was added and stirred for 5 min. The formulations are listed in Table 1. The solid content, viscosity, and pH of the adhesives was determined with reference to the GB/T 14074-2017.

The three-layer poplar plywood made in the laboratory had the dimensions of 400 mm × 400 mm. After adhesive loading (single-sided adhesive consumption: 220 g/m^2^), the plywood was assembled and cold-pressed for 10 min, placed in a drying oven at 80 °C for 1 h, and then rapidly taken out for hot-pressing at 220 °C, a pressure of 1.2 MPa, and a time of 1 min/mm. The prepared plywood was cut into 100 mm × 25 mm samples. Three plywood samples were used in each experiment. In accordance with the national standard of GB/T 17657-2013, the dry and wet bonding strengths (testing method for type II plywood) were tested, and the final strength was calculated from the average value of 24 specimens.

### 2.3. Orthogonal Experiment

Given that the bonding performance of the plywood was closely related to the hot-pressing processing parameters, the temperature, time, adhesive loading, and m(tannin):m(sucrose) were taken as four test factors and the wet bonding strength as the measurement index to design the experiment according to an orthogonal table L16(4^4^) (Table 2).

### 2.4. Insoluble Substances Rate in Cured Adhesives

The adhesives were placed in tin foil and dried in a thermostatic ventilation oven at 60~70 °C [36]. Then, the adhesive was taken out, ground and passed through a 200-mesh sieve to obtain the adhesive powders (also used to test the curing performance of the tannin–sucrose composite adhesives). 2.0 g of the adhesive powders were taken, dried in a thermostatic ventilation drying oven at 220 °C for 12 min, and then taken out and ground into powders (also used to test the chemical structure, crystallization properties and thermal performance of the tannin–sucrose composite adhesives). m0 of the sample powders were wrapped using filter paper, soaked in water at 63 °C for 6 h, and dried in a thermostatic drying oven at 120 °C. The masses of the obtained powders were calculated as m1. Finally, the insoluble content of the adhesive curing products was calculated from the average value of 8–10 samples.

### 2.5. Fourier Transform-Infrared (FT-IR) Spectrometry

The test was performed using a Varian 1000 (Varian, Palo Alto, CA, USA) IR spectrometer, in transmittance test mode with a wavenumber range of 400~4000 cm^−1^, resolution of 4 cm^−1^, scanning time of 32, indoor temperature of 22~25 °C, and a relative humidity of ≤60%. 

### 2.6. Thermogravimetric (TG) Analysis

The TG 209 F3 thermogravimeter produced by German NETZSCH was used for TG analysis under a N_2_ atmosphere at a heating rate of 10 °C/min within the range of 30–700 °C. 

### 2.7. X-ray Diffraction (XRD) Analysis

The tests were carried out using a TTR XRD (Tokyo, Japan) with a Cu target (λ = 0.154060 nm), a 2θ scanning interval of 5–80°, a step size of 0.02°, a scanning rate of 5°/min, a tube current of 120 mA, and a tube voltage of 40 kV.

## 3. Results and Discussion

### 3.1. The Effect of Pre-Drying before Hot-Pressing on the Bonding Performance of Plywood

**Figure 2:**
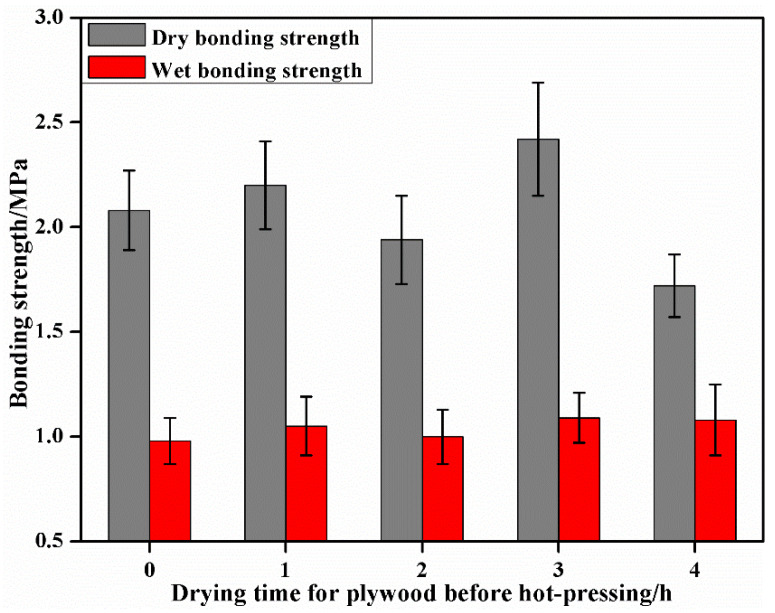
The effect of pre-drying time before hot-pressing on the bonding performance of plywood.

Figure 2 shows the effect of different pre-drying times in the oven at 80 °C before hot-pressing on the bonding performance of the plywood. The dry bonding strength and wet bonding strength of plywood without the heat treatment were 2.08 and 0.98 MPa, respectively. As the heat treatment time increased, the dry and wet bonding strengths presented an increasing trend, reaching the maximum values of 2.42 and 1.09 MPa, at 3 h with an increase of 16% and 11%, respectively. The moisture content of the plywood increased after adhesive loading. The initial moisture content was high in the plywood, which, on the other hand, led to excessive permeation of the adhesives into the wood tissues and reduced the bonding strength. On the other hand, the crosslinking degree of the adhesives was reduced when the moisture diffused outwardly during the hot-pressing, which resulted in the failure of the surface bonding interface, thus affecting the bonding performance. The bonding strength, and especially the water resistance, of the plywood was significantly improved by the heat treatments before the hot-pressing. Because wood has a porous structure, resin canals and gum canals form from the heat treatment, thus the permeability is enhanced so the adhesives can easily permeate into the wood’s surface, thus increasing the glue nails between the wood. Furthermore, the mechanical interlocking action on the wood’s surface was strengthened, thus improving its bonding strength [30]. However, the wood’s surface can be passivated with long heat-treatment times, which reduced the reactivity with the adhesives. Therefore, the heat treatment at 80 °C for 2 h was adopted for plywood preparation with tannin–sucrose adhesives in this experiment.

### 3.2. The Effect of Solid Content of Tannin–Sucrose Adhesives on the Bonding Performance of Plywood

**Figure 3:**
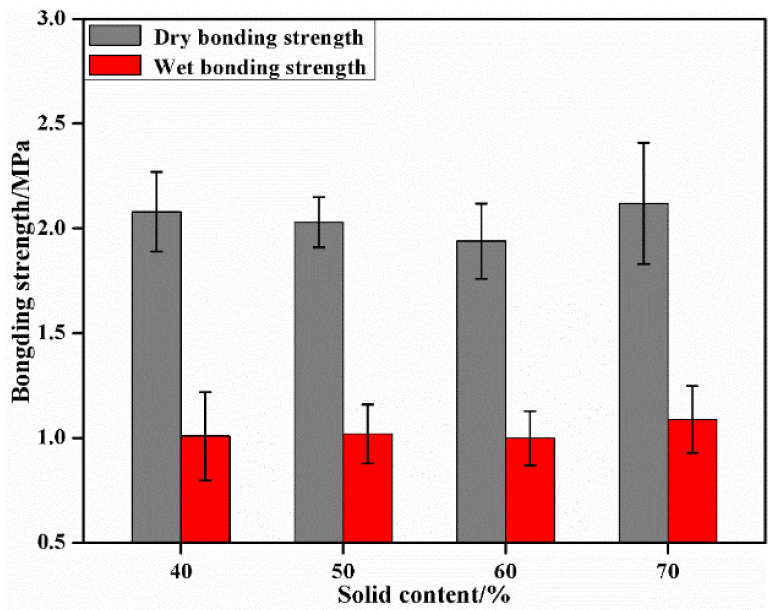
The effect of solid content of tannin–sucrose adhesives on the bonding performance of plywood.

Figure 3 exhibits the results of the bonding performance of the plywood when the solid content of tannin–sucrose adhesives was within 40–70%. Solid content, one of the important physical properties of adhesives, affects the adhesive distribution and the formation of adhesive layers during hot-pressing, thus playing a very significant role in the bonding performance of adhesives [1,5]. In the case of a low-solid content, continuous adhesive layers were not formed, and lost adhesion easily, thus leading to unstable bonding. In addition, a low-solid content also meant that more moisture needed to be removed during the hot-pressing, which posed an internal stress on the plywood and reduced its water resistance. Good bonding performance can be generally achieved only when the solid content of the adhesive reaches above 35% [37,38], but the solid content of most bio-adhesives cannot be too high due to their high viscosity. The effect of solid content on the bonding performance of the plywood was less than experimental error. However, the solid content of tannin–sucrose adhesives reached as high as 70% while not hindering adhesive application, another significant advantage of tannin–sucrose adhesives compared with soy protein-based adhesives.

### 3.3. The Effect of Mass Ratio of the Tannin to Sucrose on the Performances of Tannin–Sucrose Adhesives

**Figure 4:**
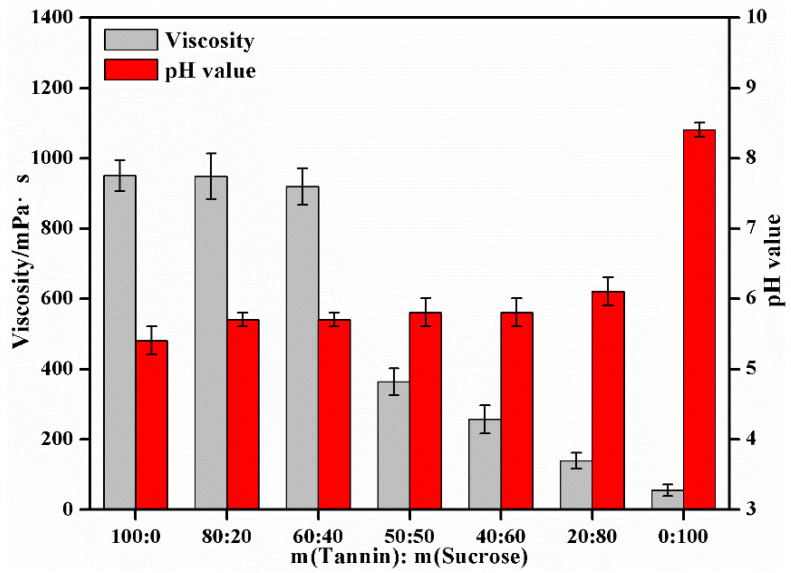
The effect of mass ratio of tannin to sucrose on the viscosities and pH of the tannin–sucrose adhesives.

**Figure 5:**
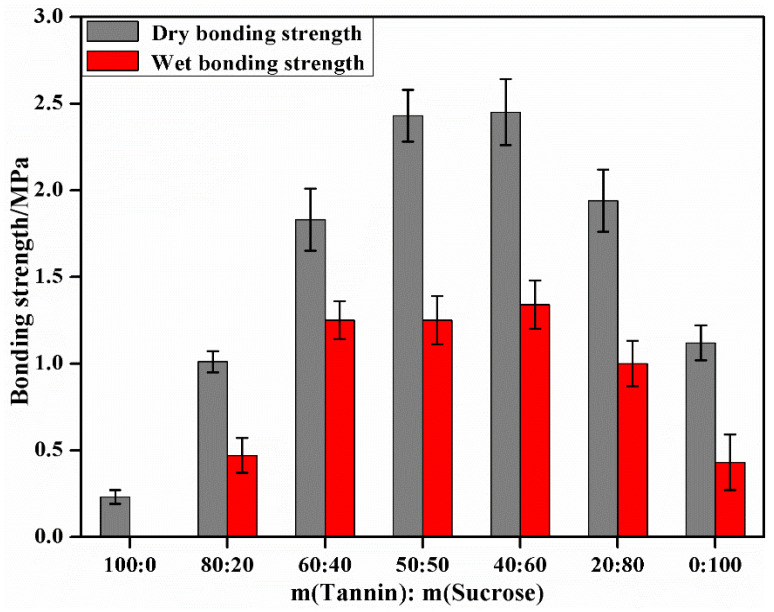
The effect of mass ratio of tannin to sucrose on the bonding performance of plywood.

Based on Section 3.1 and Section 3.2, the effects of tannin–sucrose mass ratio on the pH and viscosity of the adhesives, the water resistance of the curing products, and the bonding performance of the prepared plywood were explored under the solid content of 60%. The effects of the tannin–sucrose mass ratio on the viscosity, pH value and bonding performance of the adhesives is displayed in Figure 4 and Figure 5. Tannin, a complex mixture, has large in molecular weight with electrostatic and hydrogen bonding with resins and polysaccharides [34,35], so the viscosity of the tannin solution was large (951.1 mPa·s). Therefore, excessive viscosity was an unavoidable problem during the preparation of the wood adhesives using tannins. High viscosity resulted in poor fluidity of the adhesives, hindering adhesive application and adhesive distribution during hot-pressing and degrading the bonding performance of the prepared plywood [5,39]. Sucrose molecules are rich in hydroxyl radicals with a strong affinity for water molecules and are far smaller molecules than tannins, so the viscosity of pure sucrose solution was very small, only 55.3 mPa·s. When the m(tannin):m(sucrose) was 80:20 or 60:40, the viscosities of the tannin–sucrose adhesives were 948.9 mPa·s and 919.2 mPa·s, respectively, and this declined slowly with the increase in sucrose. When the m(tannin):m(sucrose) was 50:50, 40:60 or 20:80, the viscosities dropped sharply to 363.8 mPa·s, 257.2 mPa·s and 139.6 mPa·s, respectively, indicating that the viscosity of the tannin–sucrose adhesive system was significantly reduced by the addition of sucrose, and this was the reason for the high-solid content of the tannin–sucrose adhesives. Due to the high-solid content and low viscosity, the tannin–sucrose adhesives could meet the requirements for adhesive spraying viscosity during the preparation of particleboard and fiberboard.

Given the large molecular weight and low-crosslinking degree of the tannins, the dry bonding strength of the plywood prepared using the pure tannin adhesives was only 0.23 MPa, without water resistance. The dry bonding strength of the plywood prepared using the pure sucrose adhesive was 1.12 MPa, and the wet bonding strength was 0.43 MPa. Partial sucrose may be converted into 5-hydroxymethyl furfural (5-HMF) during hot-pressing [30,31,32], which exerted a certain crosslinking effect, but with a relatively low molecular weight. If 5-HMF alone participated in the bonding, the cohesive strength and crosslinking density of the formed adhesive layer would be low. In addition, the adhesive pH also had an important effect on the conversion efficiency of sucrose into 5-HMF, and acidic condition was more conducive to the formation of 5-HMF. There are many highly electronegative oxygen-containing groups in sucrose molecules, which can easily adsorb H^+^ in aqueous solution such that sucrose solution is slightly alkaline with a pH = 8.4, with a low sucrose conversion rate.

When the m(tannin):m(sucrose) was 80:20, the wet bonding strength was only 0.47 MPa, which was evidently an improvement compared with that of the pure tannin adhesives. This was because the amount of sucrose was relatively small in this case, and the 5-HMF that could be formed by the adhesive system was limited, leading to the limited improvement in the crosslinking degree and cohesive strength of the finally cured adhesive. H^+^ ionized by tannins provided a certain acidic environment for the adhesive system. When the m(tannin):m(sucrose) was 60:40, 50:50 or 40:60, the pH values of the adhesive system were 5.7, 5.7, and 5.8, respectively, and an acidic environment could facilitate the formation of 5-HMF. In this case, the dry bonding strength of plywood was 1.83, 2.43, and 2.45 MPa, and the wet bonding strength reached 1.25, 1.25, and 1.34 MPa, respectively, so both the dry and wet bonding strengths were much higher than 0.70 MPa (strength requirements for Type II standard of plywood in GB/T 17657-2013). This result revealed that when the additive amount of sucrose was sufficient, the tannin–sucrose adhesive system generated enough 5-HMF to crosslink with tannin during hot-pressing, and the cured adhesive showed a high-crosslinking degree and high cohesive strength, which was macroscopically manifested by a high bonding strength and water resistance.

When the m(tannin):m(sucrose) was 20:80, the dry and wet bonding strengths of the plywood were 1.94 and 1.00 MPa, respectively, both of which dropped to some extent, for the following reasons: (1) The pH value of the adhesive system started rising (pH = 6.1), and the conversion rate of 5-HMF declined; (2) the additive amount of tannin was limited, while that of sucrose was excessive, the generated 5-HMF possibly failed to completely participate in the crosslinking reaction of tannins, and the small molecular 5-HMF was dispersed in the adhesive system, thus influencing the curing of the adhesive; (3) the excessive addition of sucrose seriously reduced the system’s viscosity (139.6 mPa·s), and the permeability of the adhesive on the wood’s surface was strong, leading to a lack of adhesive on the surface and degrading the bonding performance.

**Figure 6:**
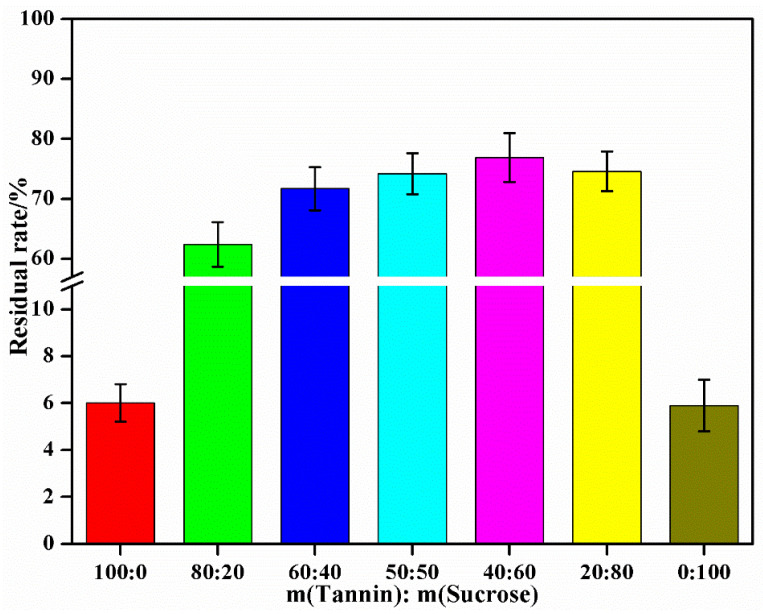
The effect of mass ratio of tannin to sucrose on residual rate of tannin–sucrose adhesives.

Figure 6 shows the results of the insoluble substance content in the cured adhesives under different tannin–sucrose mass ratios. The insoluble substance content was below 6% under both independent addition of tannin and sucrose, but when the tannin–sucrose mass ratio was 80:20 to 20:80, the insoluble substance content ranged from 62.4% to 76.9%. The insoluble substance content in the curing products of the tannin–sucrose composite adhesives significantly increased, proving the crosslinking reaction of tannin and sucrose [36]. Under the different mass ratios, the crosslinking degree and intensity of the cured adhesives were varied. To sum up, the influence of the tannin–sucrose mass ratio on the insoluble substance content was consistent with that on the bonding performances of plywood.

### 3.4. Results of Orthogonal Experiments and Analysis

Table 3 and Table 4 display the wet bonding strength of the plywood and its range and variance analysis results under different plywood preparation process conditions.

**Figure 7:**
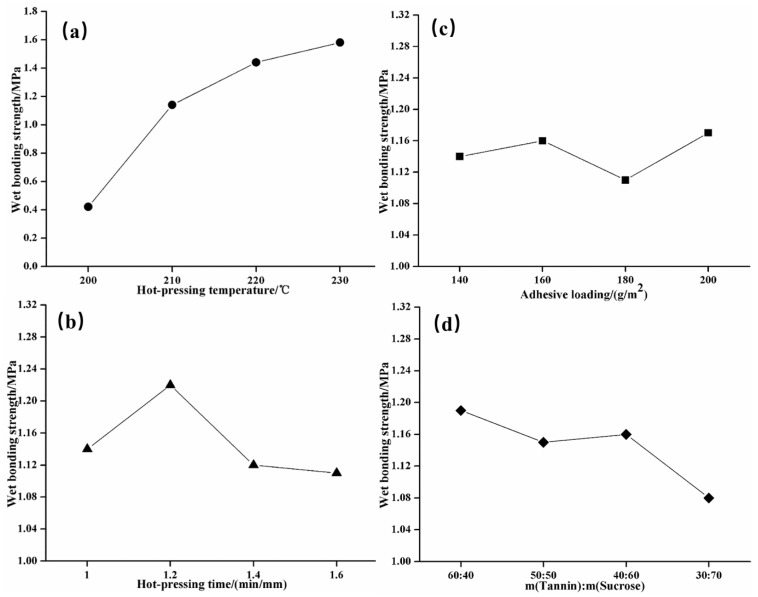
The effect of each factor of orthogonal testing on the wet bonding strength of plywood. Note: (**a**) hot-pressing time, (**b**) hot-pressing temperature, (**c**) adhesive loading, (**d**) mass ratio of tannin to sucrose.

Figure 7 presents each factor of the orthogonal tests on the wet bonding strength of the plywood. Combining Figure 7a, and Table 3 and Table 4 demonstrates that the hot-pressing temperature exerted significant influence on the wet bonding strength of the plywood. This was because the key role for the tannin–sucrose adhesive to generate a good bonding performance lies in the indirect crosslinking action of sucrose, namely, sucrose crosslinks with tannins after being converted into 5-HMF. An acidic environment and high temperatures were two key factors influencing sucrose conversion into 5-HMF, especially, the latter was critical for the formation of 5-HMF [30,31,32,40]. The bonding strength was only 0.42 MPa at a hot-pressing temperature of 200 °C, but it increased to 1.14, 1.44, and 1.58 MPa (satisfying the strength requirements for Type II plywood in GB/T 17657-2013) when the hot-pressing temperatures were 210 °C, 220 °C and 230 °C, with increases of 171%, 243%, and 276%, respectively. Therefore, the hot-pressing temperature played a decisive role in the strength performance of the tannin–sucrose adhesives. Ideal bonding strength was only obtained when the hot-pressing temperature was above 210 °C. Even though the bonding strength was higher at 220 °C and 230 °C, but a hot-pressing temperature too high easily led to plywood deformation and color change [41,42], accompanied by a greater energy consumption. Therefore, 210 °C was considered a relatively appropriate hot-pressing temperature.

Figure 7b shows the effect of hot-pressing time on the wet bonding strength of plywood. It can be seen that the bonding strength reached a maximum at a hot-pressing time of 1.2 min/mm, but declined if the hot-pressing time was continuously lengthened, demonstrating that the curing and crosslinking reactions of the tannin–sucrose adhesives was basically completed within 1.2 min/mm. Therefore, 1.2 min/mm was considered the relatively appropriate hot-pressing time of the tannin–sucrose adhesives.

Figure 7c displays the influence of adhesive loading on the wet bonding strength of the plywood. It can be seen that when the adhesive loading was 140~200 g/m^2^, the bonding strength was 1.11~1.17 MPa, so the bonding strength was influenced little by adhesive loading. The adhesive loading of 140 g/m^2^ could also meet the requirements of this experiment. However, in the process of the experiment it was found that uneven adhesive loading was occasionally caused by rapid water penetration of the adhesive. Therefore, given that adhesives should be applied to the plywood as evenly as possible, the adhesive loading of 160 g/m^2^ was deemed appropriate.

Figure 7d displays the influencing analysis of the tannin–sucrose mass ratio on the wet bonding strength of the plywood. It can be observed from Figure 7d that the bonding strength was at a maximum when the m(tannin):m(sucrose) was 60:40, slightly decreased at 50:50 and 40:60, and started dropping at 30:70. In general, the mass ratio exerted an insignificant effect on the bonding strength. Given the viscosity reduction effect of an increasing amount of sucrose, m(tannin):m (sucrose) of 60:40 was considered an appropriate.

The effects of the orthogonal test factors on the wet bonding strength of the plywood were in order of hot-pressing temperature > tannin–sucrose mass ratio > hot-pressing time > adhesive loading, among which the hot-pressing temperature had the most significant influence, while the influence of hot-pressing time, m(tannin):m(sucrose), and adhesive loading were insignificant. Plywood was prepared by the optimal process parameters, namely, hot-pressing temperature of 210 °C, time of 1.2 min/mm, m(tannin):m(sucrose) of 60:40, and adhesive loading of 160 g/m^2^. The wet bonding strength of the plywood prepared under these process parameters was 0.89 MPa, meeting the strength requirements for a Type II standard of plywood specified in GB/T 17657-2013. It even had certain wet bonding strength in boiling water (0.42 MPa), with the potential of meeting the strength requirements for a Type I standard of plywood in GB/T 17657-2013.

### 3.5. FT-IR Analysis

**Figure 8:**
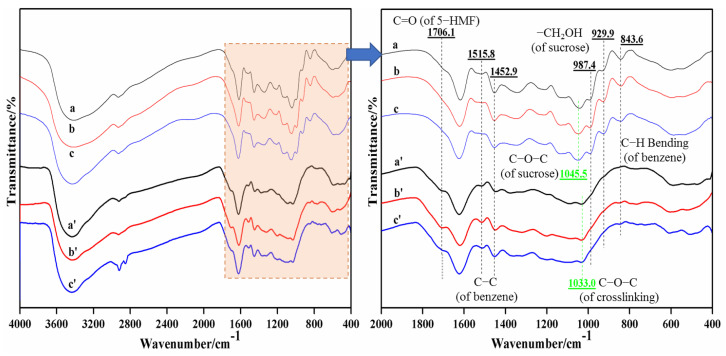
FT-IR curves of tannin–sucrose adhesives before and after curing. Note: a mass ratio of tannin to sucrose (60:40) without heat treatment, a’ mass ratio of tannin to sucrose (60:40) with heat treatment, b mass ratio of tannin to sucrose (50:50) without heat treatment, b’ mass ratio of tannin to sucrose (50:50) with heat treatment, c mass ratio of tannin to sucrose (40:60) without heat treatment, c’ mass ratio of tannin to sucrose (40:60) with heat treatment.

Figure 8 displays the FT-IR results before and after adhesive curing under different tannin–sucrose mass ratios. The tannin–sucrose adhesives (a, b and c) were in a mixed state before curing, the wavenumbers 1547.1 cm^−1^, 1515.8 cm^−1^, and 1452.9 cm^−1^ were the skeleton carbon absorption peaks on the tannin benzene ring, and the C-H bending vibration absorption peak of the tannin aromatic ring appeared at 843.3 cm^−1^. The ether bond absorption peak from the sucrose appeared at 1045.5 cm^−1^, and the hydroxymethyl absorption peaks of sucrose appeared at 987.4 cm^−1^ and 929.9 cm^−1^ [30,31,32]. The absorption peaks of the curing products of the tannin–sucrose adhesives (a’, b’, c’) at 1045.5 cm^−1^, 987.4 cm^−1^ and 929.9 cm^−1^ disappeared, indicating the depolymerization of sucrose hexatomic ring. The absorption peak at 843.3 cm^−1^ disappeared, revealing that the active hydrogen of the tannin phenol ring experienced a substitution reaction. A new C=O absorption peak was generated at 1706.1 cm^−1^, which was attributed to sucrose conversion into 5-HMF [43,44]. The C-O ether bond absorption peak at 1033.0 cm^−1^ was attributed to the product formed from the polycondensation products of 5-HMF and tannin, proving that under high-temperature conditions, sucrose was pyrolyzed and converted to form active 5-HMF to participate in the crosslinking reaction of sucrose with tannin.

Under different tannin–sucrose mass ratios, the absorption peaks of the curing products at 1706.1 cm^−1^ and 1033.0 cm^−1^ differed little in peak intensity, demonstrating that the 5-HMF formed by the adhesive system at a mass ratio of 60:40 was enough, and the yield rate of 5-HMF was not affected if the additive amount of sucrose was continuously increased.

### 3.6. XRD Analysis

**Figure 9:**
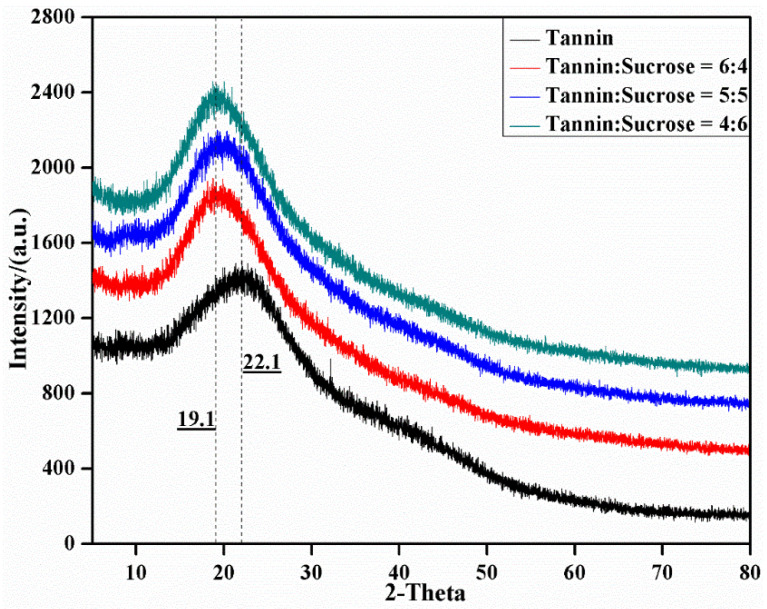
XRD curves of the curing products for the tannin–sucrose adhesives.

Figure 9 exhibits the diffractograms of the adhesives under different tannin–sucrose mass ratios. Tannins had a large wide peak at 22.1°, indicating that it was amorphous [22,23]. The tannin–sucrose adhesive showed an evident crystallization peak at 19.1° [31,32]. The degree of crystallinity reflects the ordered molecular arrangement degree. The degree of crystallinity was elevated after the tannin–sucrose crosslinking reaction, indicating a high degree of crosslinking reactions. According to MDI Jade 6 analysis, the degree of crystallinity was at a maximum at a mass ratio of 60:40, but showed a declining trend with the increase in the additive amount of sucrose. Combining the previous conclusions, 5-HMF conversion was influenced by temperature and pH of the acidic system. At a fixed temperature, the acidity was mainly derived from the tannins. With an increase in sucrose and a reduction in tannins, the weakening acidity reduced the conversion to 5-HMF. In addition, the crosslinking reaction of the adhesive system was also affected by the excessive introduction of micromolecular sucrose.

### 3.7. TG-DTG Analysis

**Figure 10:**
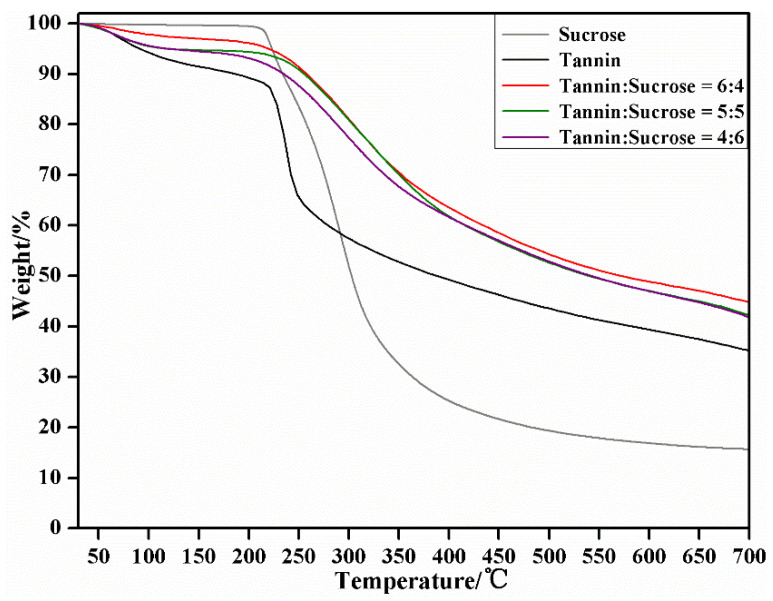
TG curves of the cured tannin–sucrose adhesives.

**Figure 11:**
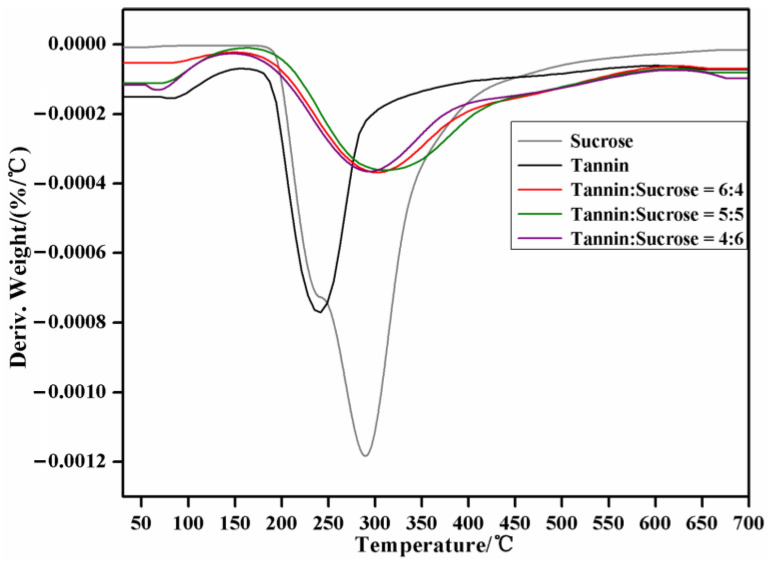
DTG curves of the cured tannin–sucrose adhesives.

Figure 10 and Figure 11 display the thermal properties (thermogravimetry, TG; differential thermal gravity, DTG) of the cured adhesives under different tannin–sucrose mass ratios. The mass loss of the five samples was all within 10% at 30–150 °C, and in this stage, the loss was mainly attributed to the evaporation of water absorbed from the air by the cured adhesives. The pure tannin adhesives from 200 °C and the tannin–sucrose adhesives from about 300 °C, experienced large weight losses, and all the residual weights were basically unchanged up to 600 °C. Within 200–600 °C, the maximum weight loss peak of the pure tannin and pure sucrose adhesives was edged, indicating their rapid weight loss and poor thermal stability, which was mainly ascribed to the low-crosslinking degree and relatively the loose structure of the pure tannin and pure sucrose adhesives. At 600 °C, the residual weight of the pure tannin and pure sucrose adhesives was 39.0% and 16.8%, respectively. This was because the molecular weight of tannin is higher than that of sucrose, so is the corresponding thermal stability. The pure sucrose adhesive had a small thermolysis peak at 240 °C, which was caused by caramel formation due to sucrose dehydration and condensation, but this peak did not last long. As the temperature rose to 290 °C, a large and edged peak appeared again, which was mainly ascribed to further carbonization and polymerization after the complete caramelization of sucrose; meanwhile, gaseous products, such as CO_2_, CO, acetic acids, and acetone as well as furfural compounds, were generated [45,46].

The TGA variation trends of the tannin–sucrose composite adhesives differed a lot from those of the pure tannin and pure sucrose adhesives, which were mainly embodied in the high thermolysis temperature, small and wide thermolysis peaks, and high residual weight, demonstrating the high thermal stability of the composite adhesives. All the TG curves of the tannin–sucrose composite adhesives showed consistent variation trends, demonstrating their similar thermal analysis courses. When the tannin–sucrose mass ratio was 60:40, 50:50 or 40:60, the maximum weight loss was at 305 °C, 312 °C, and 295 °C, respectively, all of which were higher than that of the pure sucrose adhesive at 290 °C, indicating that a thermolytic peak of sucrose degradation products and tannin crosslinking products appeared at this position, especially the crosslinking products between 5-HMF and tannins [30,31,32,43]. With the increase in sucrose and the reduction in tannins, the temperature showed a declining trend at this position, reflecting the degrading structural stability of the crosslinked products. This was because, in addition to the temperature, the acidity of the adhesive system was also very important for the sucrose conversion to 5-HMF. Tannins being of a certain acidity promoted the conversion of 5-HMF to some extent. With the declining proportion of tannins to sucrose, the acidity of adhesive system was lessened, and the conversion rate of sucrose to 5-HMF was also reduced. At 600 °C, the residual weight of the tannin–sucrose composite adhesives was 47.0~48.8%, and the residual weights were in the order of 60:40 > 50:50 > 40:60. Nevertheless, the thermolytic course and thermal stability of composite adhesives were influenced little by the different tannin–sucrose mass ratios in this study.

### 3.8. Bonding Mechanism Analysis of the Tannin–Sucrose Adhesive

**Figure 12:**
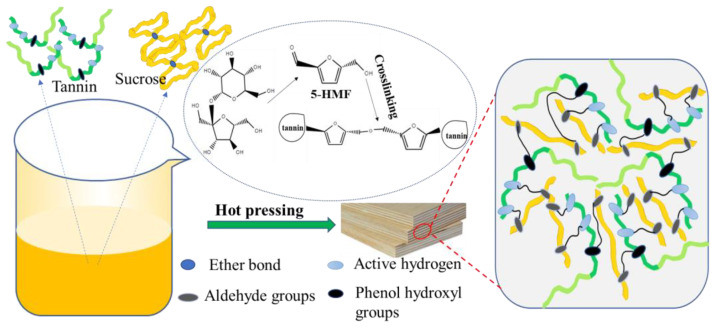
Schematic diagram of the bonding mechanisms for the tannin–sucrose adhesives.

The bonding process of the adhesives is very complicated, combining adhesive flow, wetting, diffusion, permeation, crosslinking, and curing on the wood’s surface. It could be argued that from the above results the main factors influencing bonding quality are: (1) the adhesives themselves, including their chemical composition, cohesive strength, viscosity, and solid content; (2) moisture content of the plywood; (3) the bonding process, including the hot-pressing temperature, the time, and adhesive loading. As can be seen in Figure 12, the tannin functions in the tannin–sucrose adhesive were as follows: (1) The macromolecules of tannin provided enough cohesive strength for the adhesive system [34,35]; (2) the acidity of the tannin catalyzed the sucrose conversion into 5-HMF. The sucrose functions in the tannin–sucrose adhesive were as follows: (1) A far smaller hydrophilic molecule, sucrose exerted a viscosity reducing effect on the adhesive system and facilitated the preparation of the tannin–sucrose adhesive with a high-solid content and low viscosity; (2) sucrose was converted into 5-HMF [43,47,48,49], which reacted with the tannins to act as an indirect crosslinker. Therefore, an appropriate tannin–sucrose mass ratio was key to obtain a tannin–sucrose adhesive with superior performance. Even so, the key factor for deciding whether the tannin–sucrose adhesive could achieve superior performance was the hot-pressing temperature which promoted sucrose conversion into 5-HMF and made it possible for tannin–sucrose crosslinking reactions.

The temperature needed for sucrose conversion into 5-HMF was high, so the hot-pressing temperature required by the tannin–sucrose adhesives was also high, which promoted the formation of 5-HMF and the improvement of the bonding strength, but resulted in the caramel color of the plywood’s surface and increased the energy consumption for plywood preparation. Moreover, it also increased the compression applied to the plywood and reduced the utilization rate of wood. Therefore, the emphasis of subsequent research lies on how to reduce the conversion temperature of 5-HMF so as to further lower the hot-pressing temperature.

## 4. Conclusions

Tannin and sucrose were mixed to form a composite adhesive and used to prepare plywood. The results showed the following:

(1) With a solid content of up to 70% and a low viscosity, tannin–sucrose adhesives have the potential to prepare particleboard and fiberboard.
(2) An appropriate tannin–sucrose mass ratio was key to acquiring adhesives with superior performance. Sucrose is converted into 5-HMF, which reacts with the tannins to exert an indirect crosslinking action, and the hydrophilia of sucrose had a viscosity reduction effect on the adhesive system. The macromolecular structure of tannins provided enough cohesive strength for the adhesive system, and acidic tannins could more easily promote the crosslinking reaction between tannins and sucrose.
(3) The hot-pressing temperature played a decisive role in the performance of the tannin–sucrose adhesives. The good performance of plywood could be guaranteed only when the temperature was 210 °C or above. The optimal process of plywood preparation based on tannin–sucrose adhesives is presented as follows: hot-pressing temperature of 210 °C, time of 1.2 min/mm, m(tannin):m(sucrose) of 60:40, and adhesive loading of 160 g/m^2^. The wet bonding strength of the plywood prepared under such conditions was 0.89 MPa, meeting the strength requirements for Type II plywood in GB/T 17657-2013.
(4) How to achieve good bonding performances for tannin–sucrose adhesives at a low curing temperature will be studied in subsequent research work.

## Figures and Tables

**Table 1:** The formulation of tannin–sucrose adhesives.

Samples	Tannin/g	Sucrose/g	Water/g
100:0	50	0	33.3
80:20	40	10	33.3
60:40	30	20	33.3
50:50	25	25	33.3
40:60	20	30	33.3
20:80	10	40	33.3
0:100	0	50	33.3

**Table 2:** The orthogonal experiment design.

Levels	Factors			
	Hot-Pressing Temperature/°C	Hot-Pressing Time/(min/mm)	Adhesive Loading /(g/m^2^)	m(Tannin):m(Sucrose)
1	200	1.0	140	60:40
2	210	1.2	160	50:50
3	220	1.4	180	40:60
4	230	1.6	200	30:70

**Table 3:** The results and range analysis of orthogonal experiments.

NO.	Hot-Pressing Temperature/°C	Hot-Pressing Time/(min/mm)	Adhesive Loading/(g/m^2^)	m(Tannin):m(Sucrose)	Wet Bonding Strength/MPa
1	200	1.0	140	60:40	0.38 ± 0.07
2	200	1.2	160	50:50	0.53 ± 0.02
3	200	1.4	180	40:60	0.33 ± 0.06
4	200	1.6	200	30:70	0.43 ± 0.03
5	210	1.0	160	40:60	1.25 ± 0.18
6	210	1.2	140	30:70	1.11 ± 0.15
7	210	1.4	200	60:40	1.21 ± 0.14
8	210	1.6	180	50:50	0.99 ± 0.10
9	220	1.0	180	30:70	1.36 ± 0.11
10	220	1.2	200	40:60	1.48 ± 0.11
11	220	1.4	140	50:50	1.49 ± 0.16
12	220	1.6	160	60:40	1.44 ± 0.10
13	230	1.0	200	50:50	1.57 ± 0.12
14	230	1.2	180	60:40	1.74 ± 0.15
15	230	1.4	160	30:70	1.43 ± 0.06
16	230	1.6	140	40:60	1.59 ± 0.15
K1	0.42	1.14	1.14	1.19	—
K2	1.14	1.22	1.16	1.15	—
K3	1.44	1.12	1.11	1.16	—
K4	1.58	1.11	1.17	1.08	—
R	1.17	0.10	0.07	0.11	—

**Table 4:** The variance analysis of orthogonal experiments on wet bonding strength.

Factors	Sum of Squares of Deviations (DEVSQ)	Degree of Freedom (DOF)	Mean Square Error (MSER)	Significance
Hot-pressing temperature	3.237	3	294.273	*
Hot-pressing time	0.028	3	2.545	
Adhesive loading	0.011	3	1.000	
m(tannin):m(sucrose)	0.026	3	2.364	
Error	0.012	3		

Note: * Means significance in 0.05 level.

## Data Availability

All the data is provided in the manuscript.
